# Anti-acetylated-tau immunotherapy is neuroprotective in tauopathy and brain injury

**DOI:** 10.1186/s13024-024-00733-9

**Published:** 2024-06-24

**Authors:** Celeste Parra Bravo, Karen Krukowski, Sarah Barker, Chao Wang, Yaqiao Li, Li Fan, Edwin Vázquez-Rosa, Min-Kyoo Shin, Man Ying Wong, Louise D. McCullough, Ryan S. Kitagawa, H. Alex Choi, Angela Cacace, Subhash C. Sinha, Andrew A. Pieper, Susanna Rosi, Xu Chen, Li Gan

**Affiliations:** 1 Brain and Mind Research Institute, Helen and Appel Alzheimer Disease Research Institute, Weill Cornell Medicine, New York, NY USA; 2 Weill Cornell Graduate School of Medical Sciences, Weill Cornell Medicine, New York, NY USA; 3 Department of Physical Therapy & Rehabilitation Science, Department of Neurological Surgery, University of California, San Francisco, San Francisco, CA USA; 4 Brain Health Medicines Center, Harrington Discovery Institute, University Hospitals Cleveland Medical Center, Cleveland, OH USA; 5 Institute for Transformative Molecular Medicine, School of Medicine, Case Western Reserve University, Cleveland, OH USA; 6 Department of Psychiatry, Case Western Reserve University, Cleveland, OH USA; 7 Geriatric Psychiatry, GRECC, Louis Stokes VA Medical Center, Cleveland, OH USA; 8 Department of Pathology, Case Western Reserve University, Cleveland, OH USA; 9 Gladstone Institute of Neurological Disease, San Francisco, CA USA; 10 College of Pharmacy and Research Institute of Pharmaceutical Sciences, Seoul National University, Seoul, Republic of Korea; 11 Department of Neurology, McGovern Medical School, The University of Texas Health Science Center at Houston, Houston, TX USA; 12 Department of Neurosurgery, McGovern Medical School, The University of Texas Health Science Center at Houston, Houston, TX USA; 13 Arvinas, New Haven, CT USA; 14 Department of Neurosciences, Case Western Reserve University, Cleveland, OH USA; 15 Weill Institute for Neuroscience, University of California San Francisco, San Francisco, CA USA; 16 Department of Neurological Surgery, University of California San Francisco, San Francisco, CA USA; 17 Department of Neurosciences, School of Medicine, University of California, San Diego, USA

**Keywords:** Immunotherapy, Acetylated tau, Tauopathy, TBI, Human plasma

## Abstract

**Background:**

Tau is aberrantly acetylated in various neurodegenerative conditions, including Alzheimer’s disease, frontotemporal lobar degeneration (FTLD), and traumatic brain injury (TBI). Previously, we reported that reducing acetylated tau by pharmacologically inhibiting p300-mediated tau acetylation at lysine 174 reduces tau pathology and improves cognitive function in animal models.

**Methods:**

We investigated the therapeutic efficacy of two different antibodies that specifically target acetylated lysine 174 on tau (ac-tauK174). We treated PS19 mice, which harbor the P301S tauopathy mutation that causes FTLD, with anti-ac-tauK174 and measured effects on tau pathology, neurodegeneration, and neurobehavioral outcomes. Furthermore, PS19 mice received treatment post-TBI to evaluate the ability of the immunotherapy to prevent TBI-induced exacerbation of tauopathy phenotypes. Ac-tauK174 measurements in human plasma following TBI were also collected to establish a link between trauma and acetylated tau levels, and single nuclei RNA-sequencing of post-TBI brain tissues from treated mice provided insights into the molecular mechanisms underlying the observed treatment effects.

**Results:**

Anti-ac-tauK174 treatment mitigates neurobehavioral impairment and reduces tau pathology in PS19 mice. Ac-tauK174 increases significantly in human plasma 24 h after TBI, and anti-ac-tauK174 treatment of PS19 mice blocked TBI-induced neurodegeneration and preserved memory functions. Anti-ac-tauK174 treatment rescues alterations of microglial and oligodendrocyte transcriptomic states following TBI in PS19 mice.

**Conclusions:**

The ability of anti-ac-tauK174 treatment to rescue neurobehavioral impairment, reduce tau pathology, and rescue glial responses demonstrates that targeting tau acetylation at K174 is a promising neuroprotective therapeutic approach to human tauopathies resulting from TBI or genetic disease.

**Supplementary Information:**

The online version contains supplementary material available at 10.1186/s13024-024-00733-9.

## Background

Tauopathies are a group of neurodegenerative diseases characterized by brain deposition of neurofibrillary tangles (NFTs) of tau protein (the microtubule-associated protein tau). NFTs are fundamental to both primary tauopathies, such as frontotemporal lobar degeneration (FTLD), and secondary tauopathies, such as Alzheimer’s disease (AD), traumatic brain injury (TBI), and chronic traumatic encephalopathy (CTE) [1]. Normally, tau protein binds and stabilizes neuronal microtubules. Under pathological conditions, however, hyperphosphorylated tau accumulates and forms aggregates that spread from diseased neurons to healthy neurons [2]. The extent of tau pathology closely correlates with neurodegeneration and cognitive impairment during disease progression [3], indicating that pathological tau is an important diagnostic marker and therapeutic target. Importantly, TBI specifically increases the risk of developing aging-related diseases of neurodegeneration, including AD, and tau pathology has been proposed to play a role in this phenomenon [4].

Tau undergoes various post-translational modifications, including phosphorylation, acetylation, and ubiquitination [5, 6]. We and others have previously reported that tau is aberrantly acetylated in AD brains and that hyperacetylated tau is enriched in NFTs [7–12]. Aberrant acetylation inhibits tau ubiquitination, which in turn slows tau degradation and leads to accumulation and spread of pathogenic tau, including phosphorylated tau (p-tau) [10, 13–15]. Accumulation of acetylated-tau (ac-tau) also induces mis-sorting of tau to dendrites, which impairs synaptic plasticity and spatial memory and propagates axonal degeneration [12, 16–18]. We have also reported that hyperacetylation at lysine residue 174 (K174) promotes tau accumulation and aggregation, which increases its toxicity in vivo [10, 13]. Reducing ac-tauK174 by inhibiting tau acetyltransferase p300 using salsalate reduces tau pathology and improves cognitive function in P301S tau transgenic mice, which carry a tau mutation that causes FTLD in people [13]. In addition, SIRT1, which deacetylates tau, markedly reduces propagation of tau inclusions in these same mice [15]. More recently, we reported that reducing TBI-induced neuronal tau acetylation is neuroprotective in TBI and has a protective role in AD pathogenesis following TBI [12]. Thus, acetylated tau could represent a novel target for therapeutic intervention to treat tauopathies from a variety of etiologies.

There has been considerable interest in using both active and passive immunotherapy to treat patients suffering from tauopathies. Indeed, several anti-tau antibodies have been explored in animal models [19–24], and some have moved on to clinical trials [25, 26]. Thus far, most of these efforts have targeted phosphorylated tau or conformation-specific tau. Although some antibodies reduce tau pathology and improve somatosensory functions in mice, the reported efficacy to date has been minimal and often associated with severe side effects [25]. Given the multitude of pathological forms of tau that exist in neurodegenerative disease [27], there is merit in exploring immunotherapy directed against additional disease-associated forms of post-translationally modified tau.

In this study, we explored the therapeutic potential of ac-tauK174 antibodies in PS19 mice as a function of exposure to TBI. Using newly generated mouse anti-ac-tauK174 antibodies, we detected elevated levels of ac-tauK174 in the plasma of TBI patients and brain tissue of PS19 mice after TBI. We then tested the efficacy of peripheral dosing of anti-ac-tauK174 antibodies in PS19 mice alone and in combination with TBI and observed robust neuroprotective efficacy with respect to cognitive functions, tau pathology, and microgliosis. Using single nuclei RNA sequencing (snRNA-seq), we also showed that anti-ac-tauK174 treatment rescues microglial and oligodendrocyte activation genes in PS19 mice exposed to TBI. Thus, our results suggest that antibodies targeting acetylated tau represent a promising new therapy for tau-related dementia.

## Methods

### Primary antibodies and reagents

Monoclonal antibodies were as follows: Tau-5 (AHB0042, Life Technologies, 1:10,000), MC-1 (a kind gift from P. Davies, 1:500), AT8 (MN1020, Thermo Scientific, 1:500), anti-Actin (Novus, NB100-74340), anti-GAPDH (MAB374, Millipore, 1:100k). Polyclonal antibodies were as follows: anti-human Tau (Dako, A0024, 1:100k), anti-Iba-1 (Wako, 019-19741, 1:500).

### Generation of anti-acetylated tau antibodies

Mouse monoclonal antibodies against ac-K174 tau were generated by Bristol Myers Squibb (New York, NY) (Clone 1) and Fred Hutchinson Cancer Research Center (Seattle, WA) (Clone 2). Briefly, both monoclonal antibodies were developed by screening the acetylated peptide and counter-screening on non-acetylated control peptide. Custom animal collections include TAU KO mice (#007251, Jackson), in addition to female BALB/c, CD1, and C57B6xBALB F1 strains. After splenectomy and hybridoma fusion, positive clones were validated for targeting peptide binding by ELISA and Western. Supernatants of dozens of subclones were screened for specificity against ac-K174 tau, where Clone 1 and Clone 2 were identified as ac-K174-tau-specific antibodies.

For Clone 2, female BALB/c, F1, and CD1 mice were immunized with a cocktail of synthetic peptides coupled to KLH. 10 ug/peptide was immunized using first complete and then incomplete Freund’s adjuvant on day 0, 14, and 28. On day 63, mice were immunized with 2 ug of each peptide. Spleens of 6 animals were removed and fused by electroporation 3 days later. After 1-days rest in tissue culture media, cells were plated into semi-solid media containing the peptides conjugated to phycoerythrin. Cells were cultured until colonies were observed. Colonies positive for PE were picked with a clone PIX2 colony-picking robot, deposited into 96-well plates, and screened by flow cytometry for binding to bead-conjugated peptides.

The two anti-ac-tauK175 antibodies used in this study were independently generated. Clone 1 was generated from wild-type mice before Clone 2 and was used in comprehensive animal studies. However, as Clone 1 was generated in collaboration with Bristol Myers Squibb, this limited our ability to share and utilize this clone in our studies. Clone 2 was generated afterward and was not subject to this same limitation, so we applied Clone 2 to biomarker and seeding assays to complement the Clone 1 study.

### Surface plasmon resonance

Surface Plasmon Resonance (SPR) was performed by Genscript in a conventional format. Clone 1 (Biotin-KGQANATR IPA(Ac)KTPPAPKTPPSS) and Clone 2 (Ac-CKGQANATRIPA(KAc)TPPAPKTPPSS-amide) antibodies were injected onto Series S Sensor Chip CM5 immobilized with anti-mouse antibody as Capture. Lys-Ac antigen was diluted and injected over the surface of flow cells 1 and 2 (association phase), followed by injection of running buffer (dissociation phase).

### Mice

Mice were assigned into gender- and age-matched treatment groups in a randomized manner. The sample size for each experiment was determined based on previous experience with each of the animal models used. Male and female PS19 mice at 2–3 months of age were purchased from the Jackson Laboratory and housed in a pathogen-free barrier facility at the University of California, San Francisco (UCSF) with a 12-h light/12-h dark cycle and ad libitum access to food and water. For non-TBI immunotherapy experiments, all behavior experiments were performed during daylight hours. For TBI experiments, the behavior experiments were performed during dark hours. All animal procedures were carried out under UCSF Institutional Animal Care and Use Committee–-approved guidelines.

### Surgical procedures

All animals were randomly assigned to each TBI or sham surgery group. Male and female animals were equally divided between groups. Animals were anesthetized and maintained at 2-2.5% isoflurane for the duration of surgery.

*Sham surgery.* Sham with sutures was performed as previously described [28, 29]. Briefly, animals were secured to a stereotaxic frame with nontraumatic ear bars. A midline incision was made to expose the skull and the scalp was sutured.

*Traumatic Brain Injury: Closed Head Injury*. TBI surgery was performed as previously described [29, 30]. Briefly, animals were secured to a stereotaxic frame with nontraumatic ear bars and the head of the animal was supported with foam before injury. Contusion was induced using a 5-mm convex tip attached to an electromagnetic impactor (Leica) at the following coordinates: anteroposterior, − 1.50 mm, and mediolateral, 0 mm with respect to bregma. The contusion was produced with an impact depth of 1 mm from the surface of the skull with a velocity of 5.0 m/s sustained for 300 ms. Any animals that had a fractured skull after injury were excluded from the study. Following impact the scalp was sutured. After all surgeries, the animal recovered in an incubation chamber set to 37 °C. Animals were returned to their home cage after showing normal walking and grooming behavior.

### Behavioral analysis

#### Morris water maze

Experimenters were blind to the genotypes or treatments of the mice for all behavioral analyses. The water maze consisted of a pool (122 cm in diameter) containing opaque water (20 ± 1 °C) and a platform (14 cm in diameter) submerged 1.5 cm under the water. Hidden platform training (days 1–5) consisted of 10 sessions (two per day, 2 h apart), of three trials each. The mouse was placed into the pool at alternating drop locations for each trial. A trial ended when the mouse located the platform. The maximum trial time was 60 s. Mice that failed to find the platform within 60 s were led to it and placed on it for 15 s. For the probe trial, 72 h after the final reversal training trial, mice were returned to the pool with a new drop location in the absence of a hidden platform. Performance was measured with an EthoVision video-tracking system (Noldus Information Technology). Visible platform training, where the platform was cued with a mounted black-and-white striped mast, was conducted for four trials after completion of probe trials. Swimming speed during the probe trials was recorded. The pre-established exclusion criterion is that mice that floated or did not swim would be excluded from analysis.

#### Novel object recognition

All surgical sutures were healed prior to behavioral analysis. For one week prior to behavioral analysis, animals were handled for habituation to investigators and room settings. Behaviors were performed in dark rooms during the animals’ wake cycle. All behaviors were recorded using an overhead camera. All animal behaviors were performed and videos were manually scored by investigators blinded to groups. Cortical-dependent short-term memory function was measured at three weeks post-surgery using a mouse novel object recognition assay [31]. The test environment consists of an open field arena under dim lighting. Mice were allowed to explore the arena for two 10-minute periods for two consecutive days (habituation phase). On day three (training phase), two identical objects (red Lego™ blocks) were secured to the floor in opposite corners of the arena using magnets, and mice were allowed to explore the arena and objects for 5 min. Five minutes later (testing phase), one of the objects was replaced with a novel object (orange Lego™ flower) of similar dimensions and texture. Mice were allowed to explore for 5 min. The objects and arena were cleaned with 70% ethanol between trials and animals. Trials were recorded and exploratory behavior was defined as time the animal spent directing its nose toward an object. Video analysis was performed by investigators blinded to injury and treatment. Data is expressed as discrimination index = (time with novel object − time with familiar object) / (total object exploration time). Mice that had less than 5 s of exploration time during either training or testing were excluded from analysis.

#### Hind limb extension reflex test

Hindlimb extension reflexes were evaluated according to the procedure and scoring system described previously [32]. Briefly, mice were suspended by the tail, and the degree of motor deficit was scored on a 0 to 1 scale: a normal extension reflex in both hindlimbs was scored as 1; imbalanced extension in the hindlimbs as 0.75; extension reflex in only one hindlimb as 0.5; the absence of any hindlimb extension as 0.25; and total paralysis as 0.

#### Wire hang

Muscle strength was measured by the inverted wire hang test. Each mouse was first placed with all four paws firmly grasping on a wire grid. The grid was then carefully inverted so that the mouse is hanging over a cage filled with bedding. The latency to fall was recorded. The maximum latency is 180 s. Mice were tested for a total of three trials, with 1 h between each trial.

#### Rotarod

Motor coordination was measured using a rotarod (Med Associates Inc., Vermont, USA) as previously described. The apparatus is equipped with infrared beams that automatically detect when the mouse has fallen off the rotating rod. On the first day (training), up to five mice of the same sex were simultaneously placed on the rotarod apparatus with the rod already rotating at the constant speed of 16 rpm (rotations per minute). The trial ended when the mouse fell off the rod or when 5 min elapsed. The mice were tested on three individual trials with an inter-trial interval between 15 and 20 min. On the second and third day of testing, five mice of the same sex were simultaneously placed on the rotarod apparatus with the rod rotating at an accelerating speed, from 4 to 40 rpm. The rotation speed increased by 4 rpm every 30 s. The trial ended when the mouse fell off the rod or when 5 min elapsed. The mice were given four trials each day for three days. The intertrial interval was between 15 and 20 min, and there was a 2 h interval between the AM and PM sessions.

#### Open Field

Spontaneous activity in the open field was measured with an automated Flex Field Open Field Photobeam Activity System (San Diego Instruments). Mice were tested for 15 min in a clear plastic chamber (41 cm by 41 cm by 30 cm) equipped with two 16 by 16 photobeam arrays for the detection of horizontal and vertical movements. Total movements, movements made in the center and the periphery of the arena were recorded automatically for subsequent analysis.

### Stereotaxic injection

Mice were anesthetized with 2% isoflurane by inhalation for the duration of surgery, and secured on a stereotaxic frame (Kopf Instruments). 3–4-month-old PS19 mice were injected 2 µL of 3.5 mg/ml synthetic tau fibrils (K18-P301L) stereotaxically at a rate of 0.5 µl/min into the dentate gyrus of left hippocampus. The following coordinates were used for dentate gyrus (anterior-posterior − 2.1, medial-lateral − 1.7, dorsal-ventral − 2.1).

### Termination

All mice were lethally overdosed using a mixture of ketamine (10 mg/ml) and xylazine (1 mg/ml). Once animals were completely anesthetized, the chest cavity was opened and blood was obtained by cardiac puncture. Following cardiac puncture animals were perfused with 1X phosphate buffer solution, pH 7.4 (Gibco, Big Cabin OK, -70011-044) until the livers were clear (~ 1–2 min).

### Hippocampal volume quantification and immunostaining

Investigators were blinded to the genotypes or treatment of the mice. For quantification of hippocampal volume, mice hemibrains were cut at 30 μm coronally, and all hippocampi-including sections were collected. Brain sections were mounted on microscope slides (Fisher Scientific) in an anterior-to-posterior order, starting from the section where the hippocampal structure first becomes visible (first section) to the section where hippocampal structure just disappears (last section). Mice with missing sections were excluded from the analyses, a pre-established criterion. Mounted brain sections were dried at room temperature for 24 h and stained with cresyl violet (Nissl staining). After being defatted for 15 min in 100% xylene and 10 min in 100% ethanol then rehydrated, sections were stained in 0.1% cresyl violet solution and mounted in DePeX mounting medium (VWR). Images were acquired with a Keyence BZ-9000 microscope. Hippocampal volume was estimated using ImageJ (NIH) Volumest plugin (http://lepo.it.da.ut.ee/~markkom/volumest/). 10–12 hippocampal-containing sections were typically used for each analysis. For immunostaining, floating sections were permeabilized and incubated in blocking solution (10% NGS in 0.3% Triton X-100 TBST) at room temperature for 1 h. After incubation with AT8 (MN1020, Thermo Scientific), Iba-1 (Wako), or MC1 (gift from Dr. Peter Davis), immunoreactive structures were detected with either Alexa Fluor 488– or Alexa Fluor 555–conjugated secondary antibodies (Invitrogen). After overnight incubation, the sections were incubated with secondary antibodies including Cy3-labeled donkey anti-rabbit IgG (Jackson ImmunoResearch) and fluorescein-labeled goat anti-mouse IgG (Vector Laboratories). All images were acquired by DM5000B microscope (Leica) or CSU-W1 spinning disk confocal microscope (Nikon) with 60× oil immersion objective lens and analyzed by either Micro-Manager software (UCSF) or ImageJ (NIH).

### Homogenization of cells and tissues for immunoblot analyses

HEK293T cells or mouse brain tissues were homogenized in RIPA buffer containing protease inhibitor cocktail (Sigma), 1mM phenylmethyl sulfonyl fluoride, phosphatase inhibitor cocktail (Sigma), 5mM nicotinamide (Sigma) and 1 µM trichostatin-A (Sigma). Mouse brain tissues were sonicated after homogenization. Lysates were centrifuged at 14,000 RPM at 4 °C for 15 min. Supernatants were collected and protein concentrations were determined by the BCA assay (Thermo Fisher). The same amount of protein was resolved on a 4–12% SDS-PAGE gel (Invitrogen), transferred to nitrocellulose membrane (Bio-Rad), and probed with appropriate antibodies. Bands in immunoblots were visualized by enhanced chemiluminescence (Pierce) and quantified by densitometry and ImageJ software (NIH). Representative blots from the same gel/membrane are shown and compared in the same figure. Samples from non-adjacent lanes are separated by a line.

### Human plasma study

Human plasma sample collection, inclusion criteria, and patient information are as described previously [12]. Acetylated tau was measured in human plasma as described previously [12]. Plasma was depleted of albumin and immunoglobulin according to manufacturer’s instructions (Bio-Rad Laboratories, Inc., 732–6701). In summary, aurum serum protein columns were washed two times with Aurum serum protein binding buffer to remove storage buffer. Sixty microliters of human plasma were diluted in 180 uL of Aurum serum binding protein buffer. 200 uL of the diluted plasma sample was loaded onto the resin, incubated for 15 min, and centrifuged through the column to collect the depleted plasma. The resin was washed with 200 uL of binding buffer, and eluate was added to the final depleted plasma sample. Protein concentration was measured using bicinchoninic acid assay (Thermo Scientific, A53225). For western blotting, depleted plasma was mixed with Laemmli Sample Buffer (Bio-Rad Laboratories, Inc., #1,610,737) with beta-mercaptoethanol (Bio-Rad Laboratories, Inc., #1,610,710) and boiled for 5 min. Proteins were loaded in 4–20% Criterion TGX Stain-Free gels (Bio-Rad Laboratories, Inc., #5,678,095). Total plasma proteins were visualized using ultraviolet light in the ChemiDocTM MP Imaging system (Bio-Rad Laboratories, Inc.). The Trans-Blot turbo system (Bio-Rad Laboratories, Inc.) was used to transfer proteins to 0.2 μm polyvinylidene fluoride membranes (Bio-Rad Laboratories, Inc., #1,704,157). Membranes were blocked in 5% milk in tris-buffered saline-tween 20 (TBST) for 1 h at room temperature. Membranes were then incubated with primary antibodies at 4 °C overnight. Membranes were washed in TBST (3 × 5 min) and incubated in horseradish peroxidase-conjugated secondary antibody. The BioSpectrum 810 Imaging System (UVP, Upland, CA) was used to detect blots developed in SuperSignalTM West Femto Maximum Sensitivity Substrate (Thermo Scientific, #34,096). Densitometry quantification of western blot signal was conducted by ImageJ2 version 2.9.0 software (National Institutes of Health, Bethesda, MD).

### Tau biosensor assay

Tau RD P301S FRET Biosensor cells were purchased from ATCC (#CRL-3275™). 125,000 cells were plated on PDL-coated coverslips in a 24-well plate. The following day, the media was changed, and cells were seeded with brain lysate. Brain lysate was prepared by homogenizing tissue in 10 times the volume of PBS with 0.02% NaN3, protease and phosphatase inhibitors, and de-acetylase inhibitors (5 mM nicotinamide and 1 μm trichostatin A). After homogenization, lysates were centrifuged at 21,000 g for 15 min at 4 degrees Celsius. The supernatant was collected, and protein concentration was measured using the bicinchoninic acid assay (Thermo Scientific, A53225). Lysates were diluted in PBS to a concentration of 1 mg/mL. Cells were seeded with brain lysate using Lipofectamine 3000 according to manufacturer instructions. Briefly, 7 uL of brain lysate (7 ug of protein) was mixed with OPTI-MEM, Lipofectamine 3000, and P3000 and incubated at room temperature for 20 min. The solution was added to each well in droplets. After 72 h of incubation, cells were fixed in 4% PFA, stained with DAPI, and mounted on slides with Vectashield. Images were acquired using Zeiss Axiolmager.M2 with a monochromatic digital camera (Zeiss AxioCam MRm Rev. 3). Images were analyzed using QuPath [33].

### Isolation of nuclei from frozen mouse brain tissue

The protocol for isolating nuclei from frozen brain tissue was adapted from a previous study with modifications [34]. All procedures were done on ice or at 4 °C. In brief, mouse brain tissue was placed in 1500 µl of nuclei PURE lysis buffer (Sigma, NUC201-1KT) and homogenized with a Dounce tissue grinder (Sigma, D8938-1SET) with 15 strokes with pestle A and 15 strokes with pestle B. The homogenized tissue was filtered through a 35 μm cell strainer and was centrifuged at 600 × g for 5 min at 4 °C and washed three times with 1 ml of PBS containing 1% BSA, 20 mM DTT, and 0.2 U µl − 1 recombinant RNase inhibitor. Then the nuclei were centrifuged at 600 × g for 5 min at 4 °C and resuspended in 500 µl of PBS containing 0.04% BSA and 1× DAPI, followed by FACS sorting to remove cell debris. The FACS-sorted suspension of DAPI-stained nuclei was counted and diluted to a concentration of 1000 nuclei per microliter in PBS containing 0.04% BSA.

### Droplet-based single-nuclei RNA-seq

For droplet-based snRNA-seq, libraries were prepared with Chromium Single Cell 3’ Reagent Kits v3.1 (10× Genomics, PN-1,000,268) according to the manufacturer’s protocol. cDNA and library fragment analysis were performed using the Agilent Fragment Analyzer systems. The snRNA-seq libraries were sequenced on the NovaSeq 6000 sequencer (Illumina) with PE 2 × 50 paired-end kits by using the following read length: 28 cycles Read 1, 10 cycles i7 index, 10 cycles i5 index and 90 cycles Read 2.

### Analysis of droplet-based single-nuclei RNA-seq

Gene counts were obtained by aligning reads to the mouse genome (mm10) with Cell Ranger software (v.6.1.2) (10× Genomics). To account for unspliced nuclear transcripts, reads mapping to pre-mRNA were counted by adding --include-introns flag. Cell Ranger 6.1.2 default parameters were used to call cell barcodes. We further removed genes expressed in no more than 3 cells, cells with a unique gene count over 4000 or less than 300, and cells with a high fraction of mitochondrial reads (> 1%). Potential doublet cells were predicted and removed using DoubletFinder [35] for each sample. Normalization and clustering were done with the Seurat package v4.0.0. In brief, counts for all nuclei were scaled by the total library size multiplied by a scale factor (10,000) and transformed to log space. A set of 2000 highly variable genes was identified with the FindVariableFeatures function based on a variance stabilizing transformation (vst). Principal component analysis (PCA) was done on all genes, and t-SNE was run with the top 15 PCs. Cell clusters were identified with the Seurat functions FindNeighbors (using the top 15 PCs) and FindClusters (resolution = 0.1). Uniform Manifold Approximation and Projection (UMAP) was performed with the top 15 PCs. For each cluster, we assigned a cell-type label using statistical enrichment for sets of marker genes and manual evaluation of gene expression for small sets of known marker genes. The subset() function from Seurat was used to subset oligodendrocytes and microglia, separately. Differential gene expression analysis was done using the FindMarkers() function and MAST [36].

### Statistical analysis

Data were analyzed with GraphPad Prism v.5 (GraphPad) or STATA12 (StataCorp LP). Differences between means were assessed with paired or unpaired Student’s t-test, one-way or two-way analysis of variance (ANOVA), followed by post hoc testing of all pairwise comparisons among genotypes (with Tukey-Kramer correction or Dunnett’s test for one-way ANOVA and Bonferroni correction for two-way ANOVA), or by mixed effects model, as indicated. Pearson’s correlation coefficients were used to quantify the linear relationship between two variables. The Shapiro-Wilk test of normality was applied to all data sets, and in cases where the data did not demonstrate a normal distribution, nonparametric tests were used to analyze statistical differences. The Mann-Whitney test was used for unpaired t-tests, the Wilcoxon matched pairs test was used for paired comparisons, and the Kruskal-Wallis test was used for ANOVAs. Outliers are pre-established as data outside of mean ± 2 S.D. All samples or animals were included for statistical analysis unless otherwise noted in pre-established criteria.

## Results

### Generation of new anti-acetylated tau (ac-tauK174) monoclonal antibodies

**Fig. 1:**
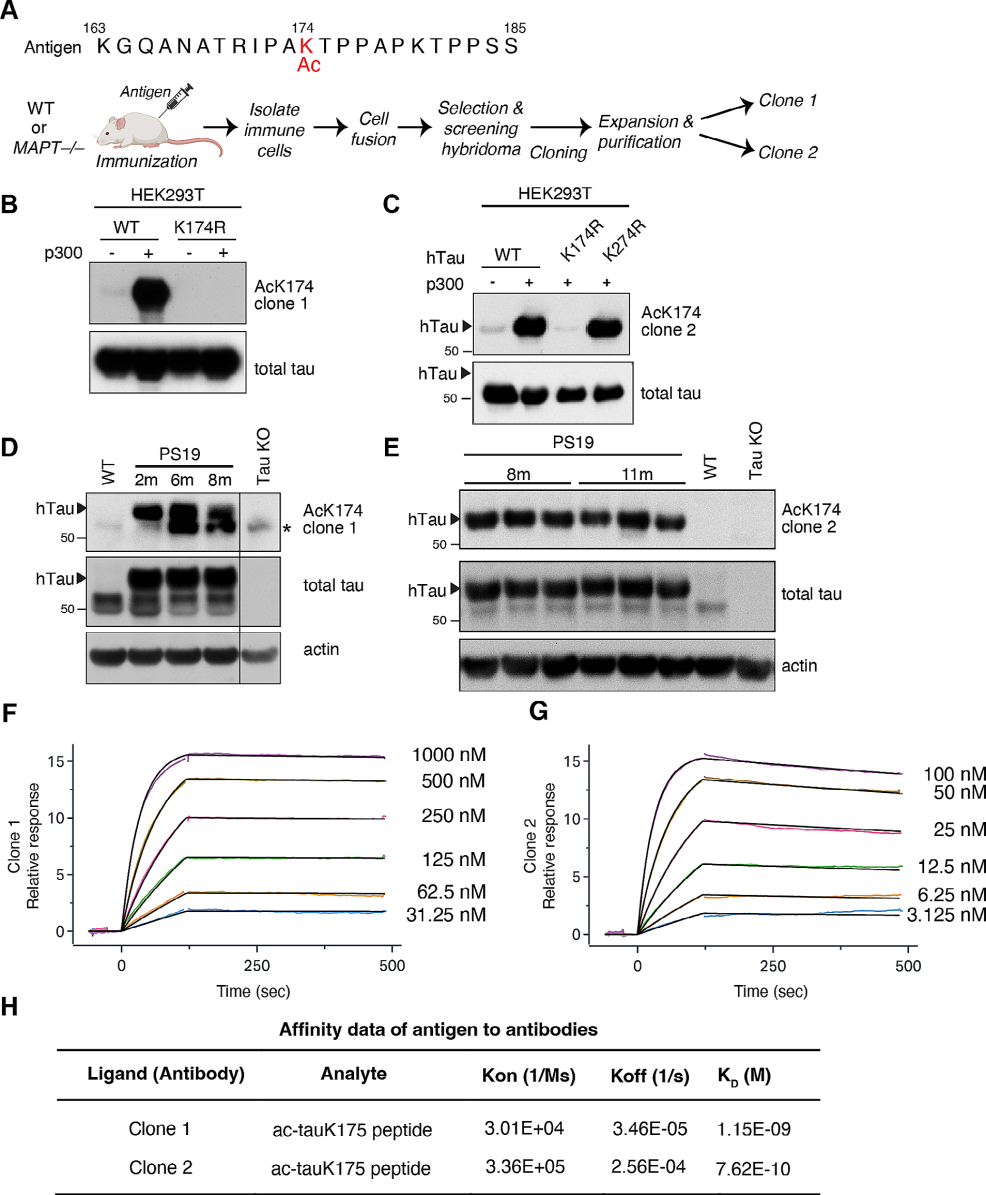
Characterization of new anti-acetylated tau (ac-tauK174) antibodies. **(A)** Sequence of the antigen peptide (human tau amino acids 163–185) and workflow used to generate monoclonal antibody against acetylated tau (Ac-K174). **(B, C)** Immunoblot of HEK293T cells transfected with p300 and WT human tau (hTau), K174R, or K274R mutant. ActauK174 signal is readily detected where WT hTau is co-transfected with p300, while K174R, but not K274R mutation blocks Clone 1 and Clone 2 signal. **(D, E)** Immunoblots of hippocampal lysate from age-ranged WT, PS19, and Tau KO mice showing that Clone 1 and Clone 2 detect ac-tauK174 immunoreactivities. Human tau (hTau, arrows) migrates at a higher molecular mass than murine tau (∼50 kDa). Asterisk indicates a non-specific band in Clone 1. **(F-H)** Specific binding affinity of Clone 1 **(F)** and Clone 2 **(G)** for ac-tauK174. Dissociation constant between clones and ligand was measured by Surface Plasmon Resonance (SPR) binding assay **(H)** using the ac-tauK175 peptide as the ligand. Kon, association rate; Koff, dissociation rate; KD, dissociation constant

To generate anti-acetylated tau (ac-tauK174) antibodies for immunotherapy in mice, we immunized monoclonal antibodies using a synthetic human tau peptide antigen (amino acids 163–185) with acetylated lysine 174 (Fig. 1A). The ac-K174 peptide was injected into either wild-type mice or tau knockout mice to enhance immune reaction. Based on ELISA-based testing of specificity and immunoreactivity, two top antibodies were selected: Clone 1 was generated in wild-type mice, and Clone 2 was generated in tau knockout (KO) mice (Fig. 1A). Both Clone 1 and Clone 2 detected strong ac-tau signal in HEK293T cells co-transfected with wild-type (WT) human tau (hTau) and p300, the acetyltransferase that acetylates tau [10, 13, 37] (Fig. 1B, C). Introducing a K174R point mutation, however, blocked Clone 1 and Clone 2 signals (Fig. 1B, C), while introducing the K274R mutation did not (Fig. 1C). We then tested these antibodies in PS19 mice, which are hTau transgenic mice that carry the P301S mutation that has been shown to cause FTLD in humans [38, 39]. Both Clone 1 and Clone 2 detected ac-tauK174 immunoreactivities in the hippocampal lysate of PS19 mice at different ages, at the same molecular weight as hTau (Fig. 1D, E). This signal was absent in WT mice and tau KO mice (Fig. 1D, E). A surface plasmon resonance (SPR) assay revealed that the dissociation constant (K_D_) for the ac-tauK174 synthetic peptide was 1.15 × 10^− 9^ for Clone 1 (Fig. 1F, H) and 7.62 × 10^− 10^ for Clone 2 (Fig. 1G, H). Together, these in vitro and in vivo data demonstrate the specificity and affinity of these new monoclonal ac-tauK174 antibodies.

### Anti-ac-tauK174 antibody rescues neurobehavioral impairment and ameliorates neuropathology in PS19 mice

**Fig. 2:**
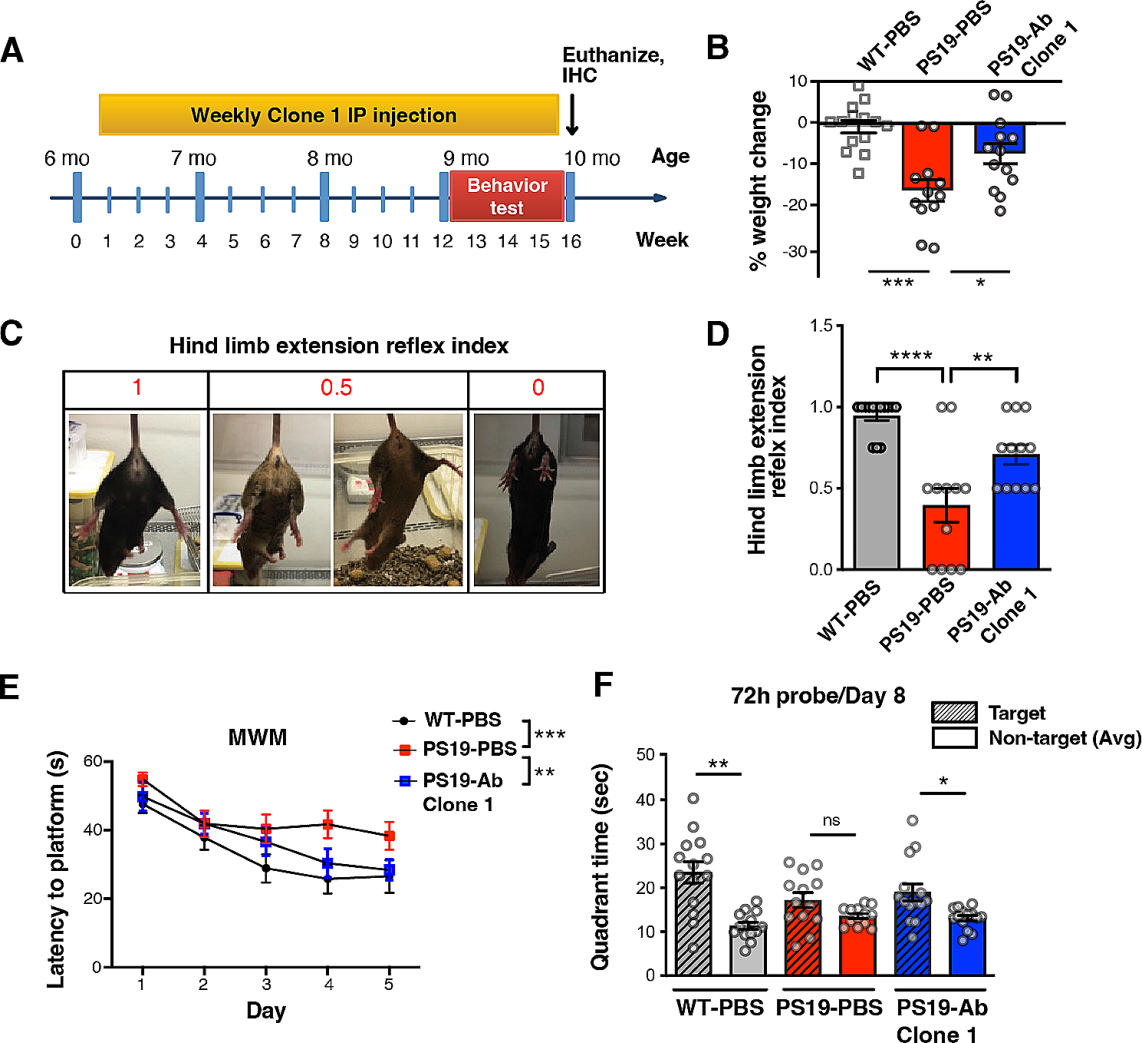
Anti-ac-tauK174 antibody Clone 1 rescues neurobehavioral impairment in P301S (PS19) mice.** (A)** Experimental timeline. At 6 months of age, male and female P301S mice were peripherally (IP) injected with Clone 1 (25 mg/kg) or vehicle (PBS). Non-transgenic (WT) littermates were injected with PBS and are included as controls. All mice received a single injection weekly for a period of 15 weeks. Behavioral analysis was performed during the last 4 weeks of treatment. Mice were sacrificed at 10 mo following the last injection. **(B)** Percentage of weight loss at the endpoint (week 15) among three groups of animals, normalized to the start point (week 1). ***p < 0.001, *p < 0.05 by one-way ANOVA, Sidak’s multiple comparison test. **(C, D)** Motor coordination impairment was measured by hindlimb extension test. Each mouse was suspended by its tail for 10 s and its hind-limb posture was scored as 1, 0.5, or 0 **(C)**. The average score for each group is presented **(D)**. ***p<0.001, **p < 0.01 by one-way ANOVA, Sidak’s multiple comparison test. **(E, F)** Spatial learning and memory were measured by Morris water maze (MWM). **(E)** Learning curve during the training phase. ***p < 0.001, *p < 0.05 by two-way ANOVA, Tukey’s multiple comparison test. **(F)** Probe trial 72 hr post-training performed on Day 8. **p < 0.001, *p < 0.05 by paired t-test. *n* = 14 per group

We next investigated the potential therapeutic effect of anti-ac-tauK174 antibody by testing Clone 1 in PS19 mice. At 25 mg/kg, Clone 1 was injected intraperitoneally (IP) in 6-month-old (mo) PS19 mice, and WT littermates, once a week for 16 weeks; PBS was injected as control. Mice at 9 months of age were subjected to behavioral and cognitive tests to assess motor and memory functions, while treatment was continued (Fig. 2A). At the end of the treatment, PS19-PBS mice exhibited significant percent weight loss (16.38%), compared to WT littermates injected with PBS, consistent with previous reports [39]. By contrast, antibody-treated PS19 mice showed significantly less percent weight loss (7.46%) (Fig. 2B), suggesting an overall positive effect on animal health.

Next, we assessed motor strength and coordination through the hindlimb extension reflex test. Here, WT mice responded to tail suspension by immediately extending both hindlimbs, which is considered normal behavior (Fig. 2C, left). As the disease progressed, PS19 mice exhibited abnormally clasped hindlimbs when suspended by the tail (Fig. 2C, right). Intermediary phenotypes ranging from normal to abnormal (0.75, 0.5, 0.25) were scored [32]. The abnormal clasping behavior was prevented when PS19 mice were treated with the anti-ac-K174 antibody (Fig. 2D). Next, we assessed spatial learning and memory by Morris water maze. In this test, mice are trained to find a platform hidden under opaque water. The time required to find the platform across multiple days is a direct measure of learning. In the training period, PBS-treated PS19 mice found the escape platform more slowly than PBS-treated WT mice (Fig. 2E). PS19 mice treated with the anti-ac-K174 antibody, however, located the escape location more quickly, indicating improved learning (Fig. 2E). At 72 h post-training (Day 8), spatial memory was assessed by removing the escape platform and measuring the time mice spent searching in its previous location, termed the target quadrant. WT-PBS mice spent more time in the target quadrant, whereas PS19-PBS mice showed no preference for this region (Fig. 2F), indicating impaired memory. Treatment with the anti-ac-K174 antibody prevented this memory impairment in PS19 mice (Fig. 2F). Importantly, there was no significant difference in swim speed between the groups (Sup. Fig. 1A). Additional behavioral analyses were performed, including the wire hang assay, which revealed no significant differences in latency to fall between groups (Sup. Fig. 1B). PS19 mice experienced significant absolute weight loss compared to WT mice at Week 13, which was not alleviated by antibody treatment (Sup. Fig. 1C). Notably, antibody-treated PS19 mice had lower weights at the beginning of the experiment compared to the other groups (Sup. Fig. 1D). In the rotarod assay, mice that received antibody treatment exhibited a significant increase in latency to fall in the last trial on the 3rd day of measurements (Sup. Fig. 1E). The open field assay demonstrated no significant difference in total and periphery movement; however, an increase in center movement was observed in PS19 mice compared to WT mice, regardless of antibody treatment (Sup. Fig. 1F).

**Fig. 3:**
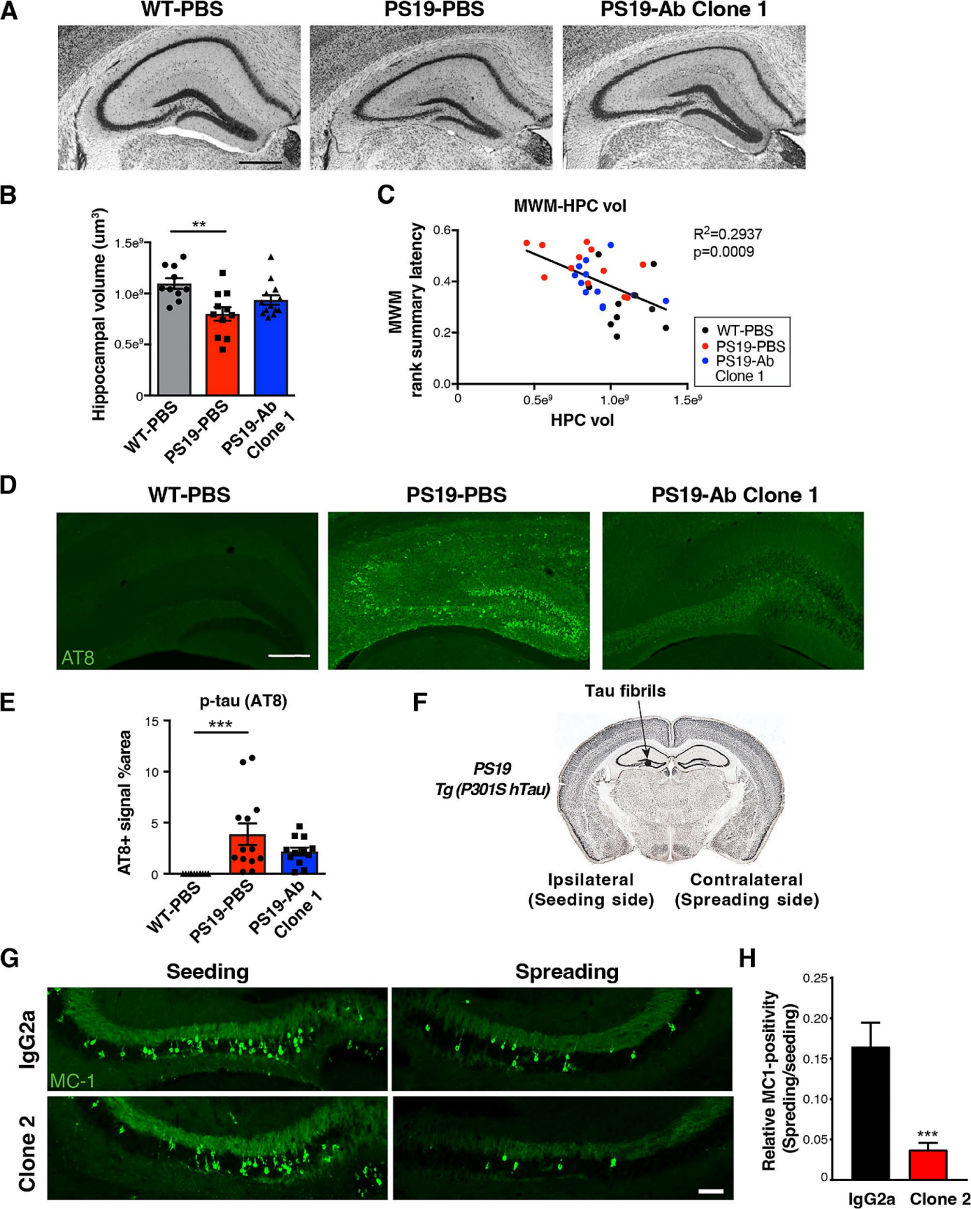
Anti-ac-tauK174 antibody ameliorates tau pathology and neurodegeneration in PS19 mice. **(A)** Representative Nissl-staining showing hippocampal morphology of WT mice treated with PBS, PS19 mice treated with PBS, and PS19 mice treated with Clone 1 antibody. Scale bar: 500 µm. **(B)** Quantification of hippocampal volume. **p<0.01 by one-way ANOVA, Kruskal-Wallis test. **(C)** Pearson correlation analysis of Morris water maze performance score (rank summary latency during learning) and hippocampal volume. **(D)** Representative immunohistochemistry staining of p-tau (AT8) in the hippocampi. Scale bar: 250 µm. **(E)** Quantification of hippocampal AT8-positive area. ***p<0.001 by one-way ANOVA, Kruskal-Wallis test. **(F)** Schematic diagram showing the injection of tau fibrils to the hippocampus of PS19 mice induces spreading of tau pathology from the ipsilateral (seeding) side to the contralateral (spreading) side of the brain. **(G)** Representative immunostaining showing MC1-positive tau inclusions in the seeding side and the spreading side after treatment with IgG2a or Clone 2 antibody. Scale bar: 100 µm. **(H)** Quantification of relative MC1-positive signal in IgG2a and Clone 2 treated groups, normalizing the spreading side to the seeding side. n = 5 mice for both groups. ***p<0.001, STATA mixed model

The neuropathology of these animals was examined at 10 months of age. PS19-PBS mice showed significant hippocampal volume loss compared to WT-PBS mice (Fig. 3A, B). Anti-ac-K174 antibody (Clone 1) treatment yielded a trend towards reduced neuronal atrophy (Fig. 3A, B, *p* = 0.09) and decreased accumulation of pathological p-tau (S202/T205, AT8) deposition in the hippocampus (Fig. 3D, E, *p* = 0.08). Correlation analysis of the relationship between pathology and behavioral phenotypes was then conducted via rank-summary score method, with raw scores replaced by quantile scores for each trial and then averaged for each subject [40]. This showed that the rank summary score based on latency in the learning phase of Morris water maze of individual animals negatively correlates with hippocampal volume (Fig. 3C). Taken together, these data indicate that anti-ac-tauK174 immunotherapy reduced tau-induced motor and cognitive impairment.

### Anti-ac-tauK174 antibody reduces fibril-induced tau spreading in PS19 mice

To investigate whether anti-ac-tauK174 antibody could affect pathological tau spreading, we next tested a second ac-K174 antibody (Clone 2) in a fibril-induced tau spreading model in vivo, in which inoculation of exogenous tau seeds induced profound tau spread in 3.5 mo PS19 mice within one month [41]. K18-P301L tau fibrils were injected in the dentate gyrus of the hippocampus of PS19 mice (AP=-2.5, ML = 2, DV = 1.8) to induce tau spreading (Fig. 3F). Clone 2 and control mouse IgG2a were IP injected weekly at 25 mg/kg for 4 weeks. Injection of tau fibrils induced tau aggregates (MC1-positive) that spread to the contralateral hippocampus within 4 weeks (Fig. 3G). Clone 2 treatment significantly reduced the amount of aggregated tau in the contralateral side, providing evidence that anti-ac-tauK174 immunotherapy reduces tau spreading in vivo (Fig. 3G, H).

### Anti-ac-tauK174 immunotherapy prevents TBI-induced neuropathology in PS19 mice

**Fig. 4:**
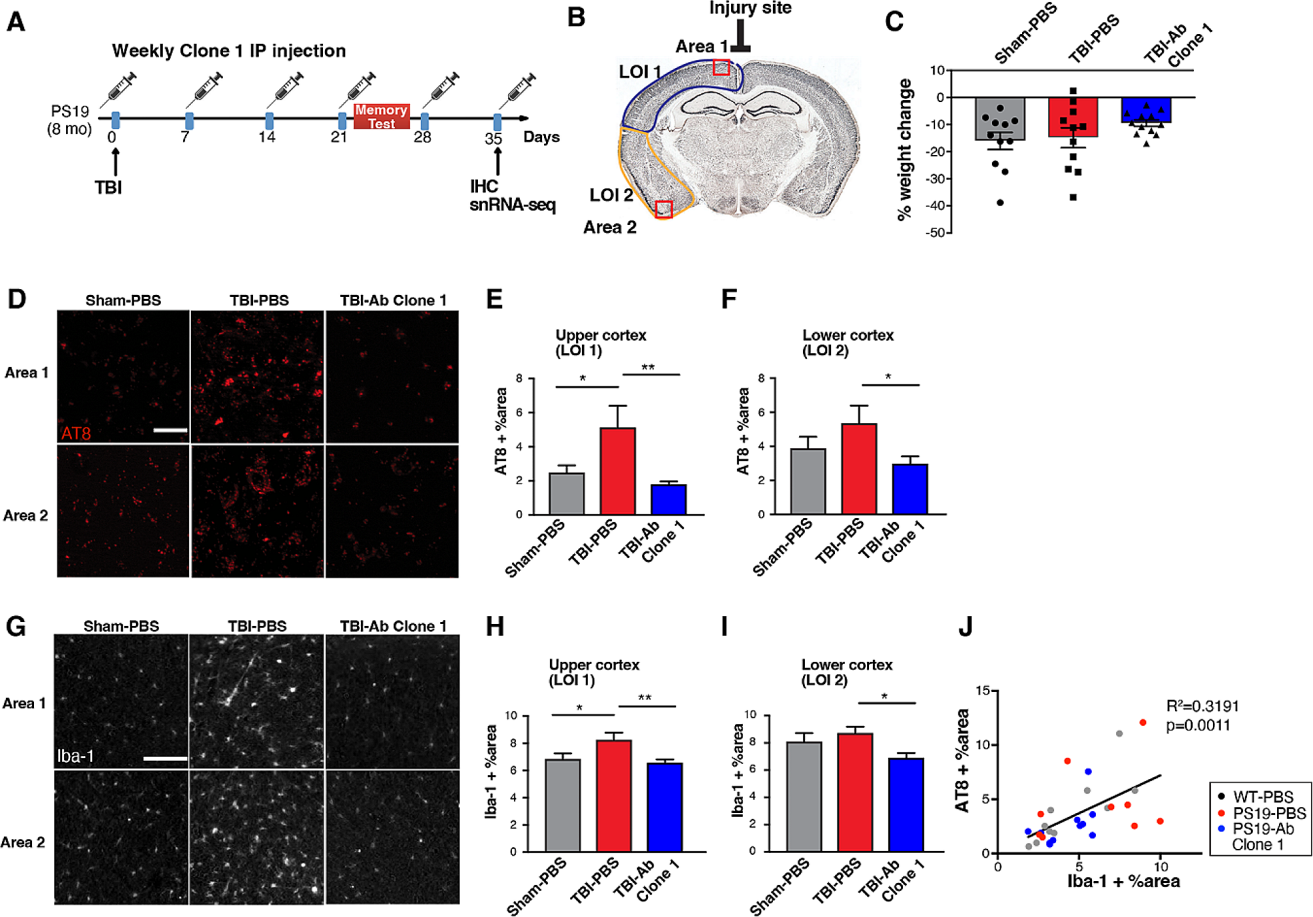
Anti-ac-tauK174 treatment ameliorates behavioral impairments and neuropathology in P301S mice with TBI. **(A)** 8-month PS19 mice were exposed to sham or traumatic brain injury (closed head concussive) surgery. One day prior to trauma surgery animals received PBS or Clone 1 administered intraperitoneally (IP). Treatment continued weekly throughout the duration of experimentation until termination 5 weeks post-injury. **(B)** Schematic diagram showing the injury site and areas of pathology analysis. **(C)** Percentage of weight loss at Day 28 among three groups of PS19 animals showing a trend of reduction in mice with TBI and treated with antibody. **(D, G)** Representative immunohistochemistry staining of AT8-positive p-tau **(F)** and Iba-1 **(I)** in the upper (LOI 1) and lower (LOI 2) cortex of PS19 mice with sham surgery or TBI, treated with PBS or Clone 1 antibody. Scale bar: 100 µm. **(E, F, H, I)** Quantification of AT8 positive % area **(E, F)** and Iba-1 positive % area **(H, I)** in upper and lower cortex of the three sections adjacent to the injury site. **p<0.01, *p<0.05 by one-way ANOVA, Tukey’s multiple comparison test. **(J)** Pearson correlation analysis of AT8-positive p-tau levels (by western blot) and Iba-1 signal in the lower cortex. n = 12 per group

TBI has been associated with exacerbated tau pathology [42], and ac-tau was recently established as the first blood biomarker of post-TBI neurodegeneration in both mice and humans that directly reflects the abundance of a therapeutic target in the brain [12]. We therefore investigated whether anti-ac-tauK174 immunotherapy could prevent trauma-induced neuropathological changes, transcriptomic alterations, and deficits in memory function (Fig. 4A).

PS19 male and female mice at 8 months of age were subjected to TBI or sham injury, and treated with Clone 1 or PBS via IP injection started one day prior to surgery and continued weekly for 5 weeks (Fig. 4A, B). Clone 1 treatment in PS19 mice produced a trend of reduced weight loss compared to both sham and TBI-PBS treated groups (Fig. 4C). We examined neuropathological changes in these animals, where PS19 mice subjected to TBI showed increased AT8-positive phosphorylated-tau (p-tau) deposition in the cortex compared to sham group (Fig. 4D-F). We next assessed microgliosis in these animals by immunohistochemistry for Iba-1. As expected, TBI mice showed increased Iba-1 signal in the cortex (Fig. 4G, H). Anti-acK174 antibody treatment, however, significantly reduced Iba-1 signal (Fig. 4G-I), and this signal positively correlated with AT8 signal in the lower cortices (Fig. 4J).

### Single nuclei RNA-seq reveals microglial activation and oligodendrocyte myelination impairment in TBI are rescued by anti-ac-tauK174 immunotherapy

To characterize the therapeutic effects of anti-ac-tauK174 (Clone 1) immunotherapy in PS19 mice subjected to TBI at the transcriptomic level, we performed single nuclei RNA-seq (snRNA-seq) using cortical tissues from 6 to 7 mo PBS-injected (PS19-Sham-PBS), TBI + PBS-injected (PS19-TBI-PBS), and TBI + Clone1-injected (PS19-TBI-Ab) PS19 transgenic male mice and WT littermates from Fig. 4. Following an established snRNA-seq protocol [34], we sequenced 120,090 nuclei from all four conditions (*n* = 4 per condition) (Sup. Fig. 2A). After removal of potential multiplets using DoubletFinder [35] and filtering for low-quality nuclei determined by thresholding gene counts, UMI counts, and percentage mitochondrial genes per nuclei (Sup. Fig. 2B-D), we selected 99,168 nuclei for downstream analysis. Using reference gene markers for annotations, we identified major cell types in the brain that were similarly represented within each group and individual mouse (Sup. Fig. 2E-G).

**Fig. 5:**
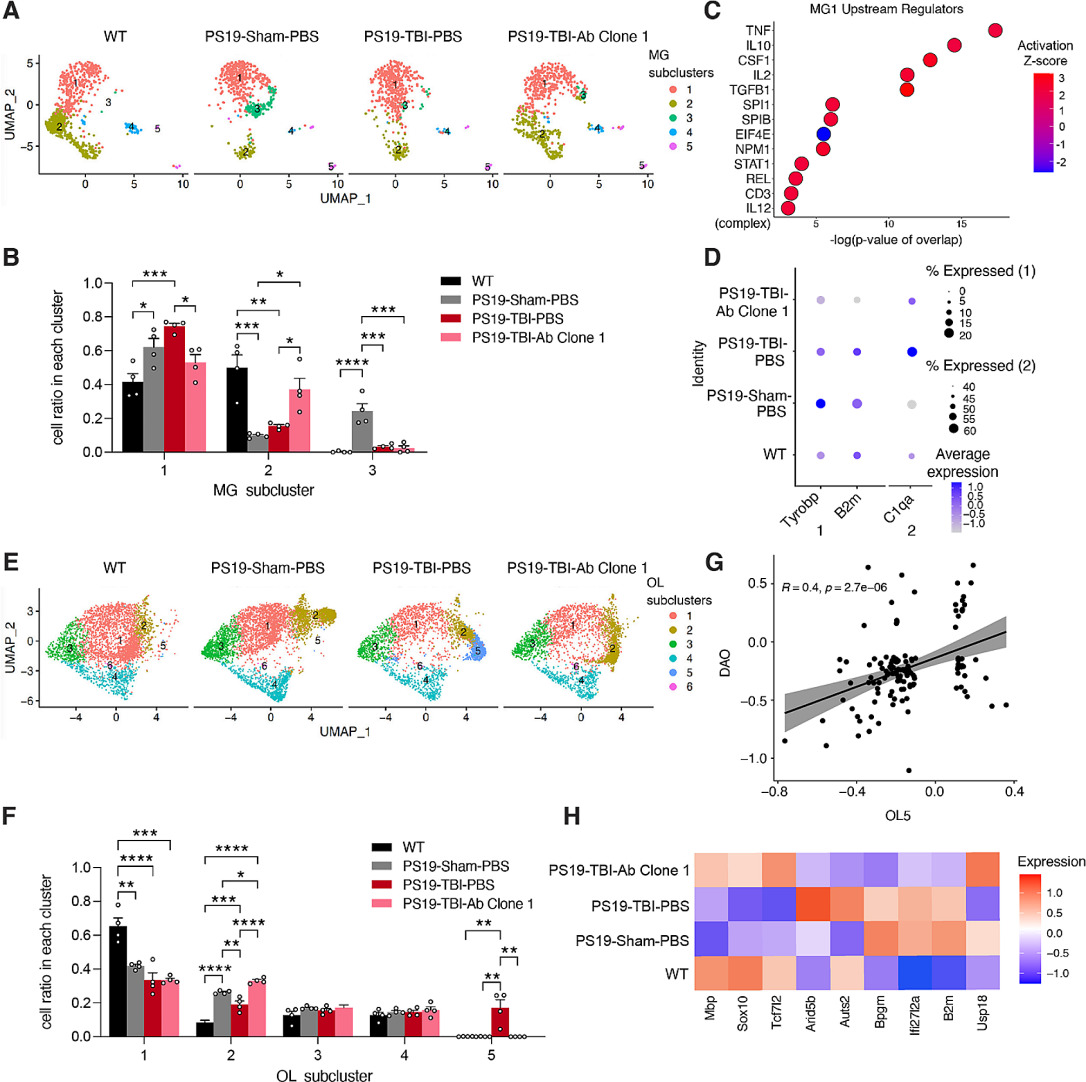
snRNA-seq reveals microglial activation and oligodendrocyte myelination impairment in TBI are rescued by anti-ac-tauK174 immunotherapy. **(A)** Subcluster analysis of microglia (resolution 0.15) between conditions: WT, PS19-Sham-PBS, PS19-TBI-PBS, and PS19-TBI-Ab Clone 1. **(B)** Bar graph of cell ratio per condition within each microglia cluster. ****p < 0.0001, ***p < 0.001, **p < 0.01, and *p < 0.05 by one-way ANOVA with Tukey’s multiple comparisons correction within each subcluster. **(C)** Ingenuity Pathway Analysis (IPA) upstream regulators of MG1 markers. **(D)** Percentage and average expression levels of disease-associated microglia (DAM) genes (Tyrobp, B2m), and cytokine gene (C1qa) across 4 conditions; scale, log2 fold-change. **(E)** Subcluster analysis of oligodendrocytes (resolution 0.2) between conditions. **(F)** Bar graph of cell ratio per condition within each oligodendrocyte cluster. ****p < 0.0001, ***p < 0.001, **p < 0.01, and *p < 0.05 by one-way ANOVA with Tukey’s multiple comparisons correction within each subcluster. (G) Simple linear regression analysis with standard error showing a positive correlation between OL5 markers and disease-associated oligodendrocyte markers (DAO) (R = 0.4, *p* = 2.7e-6). **(H)** Heat map of average expression levels of myelination-related genes (Mpb, Sox10, Tcf7l2), IFN-gamma hallmark genes (Arid5b, Auts2, Bpgm, Usp18), and IFN-responsive oligodendrocyte (IRO) markers (Ifi27l2a, B2m) across 4 conditions; scale, log2 fold-change

We subset and reclustered 2,718 microglial cells (841 from WT, 630 from PS19-Sham-PBS, 562 from PS19-TBI-PBS, 685 from PS19-TBI-Ab) to investigate how anti-ac-tauK174 altered microglial activation states in PS19 mice with TBI. The microglial population from the four conditions exhibited five clearly defined subclusters (Fig. 5A, Sup. Table 1). Analyses of the distribution of these five subclusters revealed that bulk sequencing of TBI-specific microglia dominated the composition of subcluster 1 (MG1) and that immunotherapy significantly reduced MG1 cell ratio to levels similar to WT and PS19-Sham-PBS mice (Fig. 5B). Ingenuity Pathway Analysis (IPA) revealed that many upstream regulators of MG1 were associated with pro-inflammatory immune responses, including cytokines (*IL2*, *IL12, TNF, IL10*) and transcription regulators (*SPIB, SPI1, REL, STAT1*) (Fig. 5C).

Microglia reactivity is a pathological hallmark of TBI, with chronic microglial activation having been reported in human patients after moderate-to-severe TBI [43, 44]. We previously reported a mixed microglial phenotype following TBI in which reactive microglia simultaneously express pro-inflammatory and anti-inflammatory markers [45]. Here, we compared the expression levels of disease-associated microglial (DAM) genes between conditions by pseudo-bulk analysis. Expression levels of *Tyrobp*, *B2m*, and *C1qa* were elevated in PS19 mice in both TBI and sham-injury conditions, and were lowered by antibody treatment (Fig. 5D). Thus, treatment with anti-ac-tauK174 antibody blunted pathological microglia reactivity.

TBI causes cerebral demyelination and impairment of remyelination mechanisms through secondary oligodendrocyte dysfunction [46], which can lead to progressive axonal degeneration after TBI and contributes to neuropsychiatric impairment and chronic neuronal cell death [47–50]. We therefore subset and reclustered 13,761 oligodendrocytes (3,395 from WT, 4,555 from PS19-Sham-PBS, 2,894 from PS19-TBI-PBS, 2,917 from PS19-TBI-Ab) to investigate whether anti-ac-tauK174 altered oligodendrocyte-mediated myelination in PS19 mice with TBI. The oligodendrocyte population from the four conditions exhibited six clearly defined subclusters (Fig. 5E, Sup. Table 2), and cluster 5 (OL5) was unique to PS19-TBI-Ab brains (Fig. 5F). We compared OL5 markers to a recently characterized disease-associated oligodendrocyte (DAO) gene signature from a meta-analysis of AD and multiple sclerosis single-cell RNA-seq (scRNA-seq) datasets [51]. We found there was a strong positive correlation (*R* = 0.4, *p* = 2.7e-6) between the cluster-agnostic gene markers associated with DAOs and OL5 markers (Fig. 5G), suggesting OL5 is comprised of oligodendrocytes with a disease-associated activation state. Thus, anti-ac-tauK174 treatment significantly reversed the TBI-induced states enriched with DAOs. We next compared the expression levels of established myelination-related genes between conditions by pseudo-bulk analysis (Fig. 5H). Antibody treatment in TBI restored the low expression level of myelin basic protein (*Mbp*) to normal WT levels, indicating protection from TBI-induced axonal demyelination. Levels of both *Sox10* and *Tcf7l2*, which cooperate during oligodendrocyte maturation to promote myelination [52], were also decreased by TBI and rescued by anti-ac-tauK174 immunotherapy.

Proinflammatory cytokines, including interferon (IFN) gamma, have been reported as key mediators of neuroinflammation in both tauopathy and TBI [53, 54]. Type I IFNs also contribute to aberrant inflammatory responses in tauopathy and after TBI [55–57]. *Arid5b, Bpgm, Auts2*, and *Usp18* are human hallmark IFN gamma genes [58]. A recent transcriptomic characterization of aged oligodendrocytes using scRNA-seq revealed a small IFN-responsive oligodendrocyte subpopulation (IRO) which was characterized by the expression of several IFN response-related genes, including *Ifi27l2a* and *B2m* [59]. PS19-TBI oligodendrocytes exhibited higher levels of IRO genes (*Bpgm, Ifi27l2a, B2m*) and IFN-gamma genes (*Arid5b, Auts2)*, which were all reduced with anti-ac-tauK174 treatment (Fig. 5H). Usp18 is a negative regulator of IFN and its deletion causes myelin pathology [60, 61]. Expression levels of *Usp18* are increased with anti-ac-tauK174 treatment after a TBI-induced depletion (Fig. 5H).

Together, expression levels of these markers indicate that anti-ac-tauK174 rescues TBI-induced transcriptomic shifts in oligodendrocyte function and activation. This transcriptomic profiling of the PS19-TBI model supports the therapeutic potential of anti-ac-tauK174 as a treatment for restoring oligodendrocyte remyelination capabilities and ameliorating disease-associated microglial responses induced by TBI.

### Anti-ac-tauK174 treatment ameliorates behavioral impairments and reduces tau seeding in vitro

**Fig. 6:**
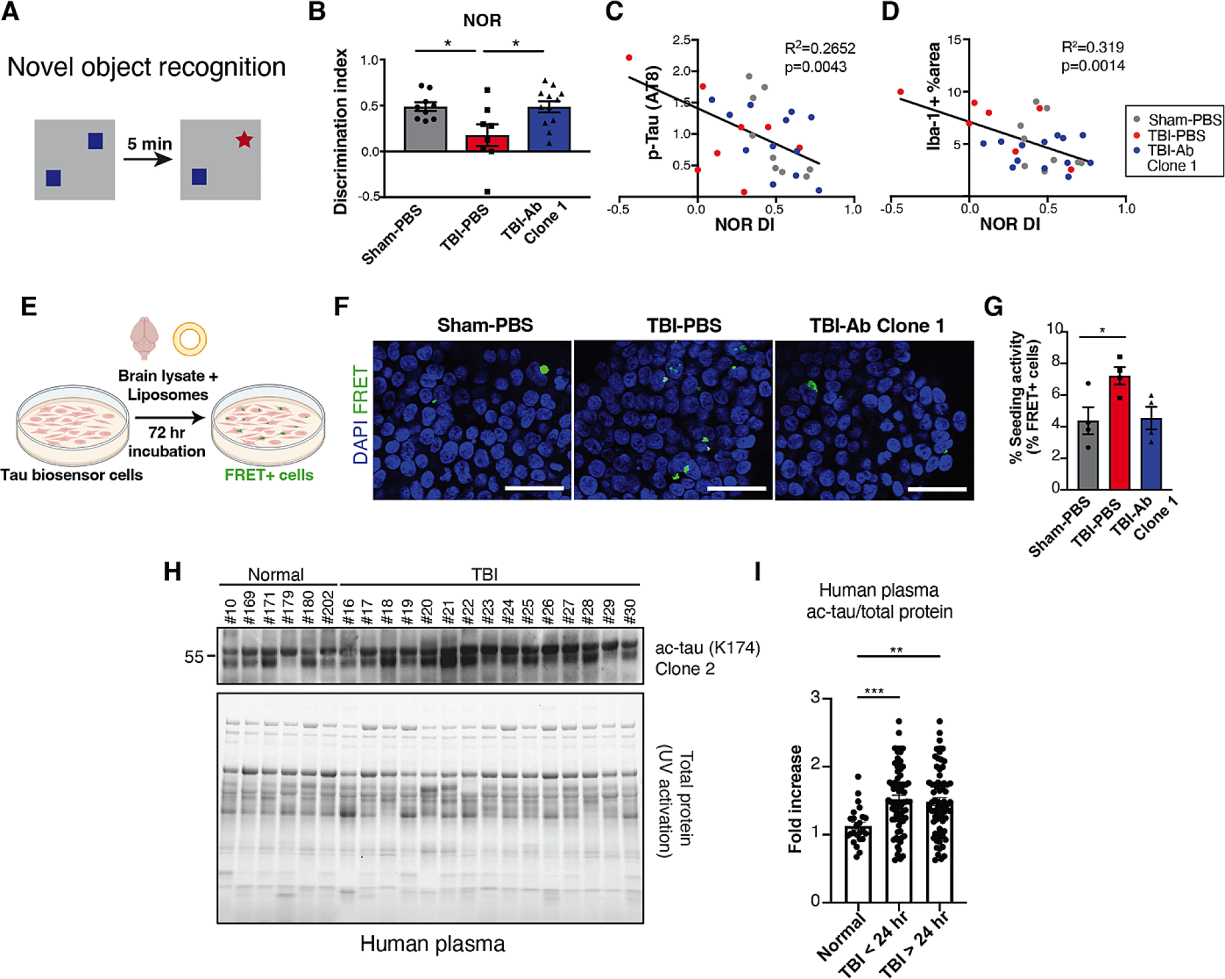
Anti-ac-tauK174 treatment ameliorates behavioral impairments and reduces tau seeding in vitro. **(A, B)** Trauma-induced memory deficits were measured by novel object recognition (NOR) test. Three weeks post-injury mice were exposed to two identical objects, five minutes later one of the objects was replaced with a new object (A). Memory deficits were calculated by decrease in time spent with the novel object graphed as a discrimination index (B). *p< 0.05 by one-way ANOVA, Sidak’s multiple comparison test. n=9 (Sham-PBS), 8 (TBI-PBS), 12 (TBI-Ab Clone 1). **(C, D)** Pearson correlation analysis of NOR discrimination index (DI) and AT8-positive p-tau levels (by western blot) (C), and Iba-1 signal (D) (by western blot). n=12 per group. **(E)** Tau RD P301S FRET Biosensor cells were treated with brain lysates and liposomes, incubated for 72 hours, and imaged to assess tau seeding activity by measuring % FRET+ cells. **(F, G)** Representative immunocytochemistry staining (D) and quantification of % CFP/YFP FRET cells (E) after incubation with PS19 mouse lysate. *p<0.05 by one-way ANOVA, Tukey’s multiple comparison test. n=4 (Sham-PBS), 4 (TBI-PBS), 4 (TBI-Ab Clone 1). Scale bar: 50 µm. **(H)** Representative immunoblot of acetylated tau (K174) (Clone 2) in control (Normal) and TBI human plasma samples. **(I)** Quantification of plasma ac-tau (K174) levels normalized by total protein levels. The mean level of ac-tau (K174) was significantly higher in the TBI cohort at 24 h in comparison to the controls (1.57 ± 0.48 versus 1.13 ± 0.27,***p< 0.001). n=24 (normal), 44 (TBI),**p< 0.01 by one-way ANOVA, Dunnett’s multiple comparison test

We investigated whether anti-ac-tauK174 (Clone 1) immunotherapy could prevent trauma-induced deficits in memory function in TBI-subjected WT and PS19 mice from Fig. 4. To test for cortical-dependent short-term memory, we utilized the novel object recognition (NOR) task 3 weeks post-injury. In this task, animals were exposed to two identical objects, and then 5 min later one of the objects was replaced with a novel object (Fig. 6A). The animal’s preference for the novel object (discrimination index, as measured by times spent with each object) provides a measure of short-term recognition memory because mice are inherently more interested in a new object. Sham animals spent more time exploring the novel object, which indicated intact short-term memory for the objects to which they had previously been exposed (Fig. 6B). TBI mice, however, displayed significantly impaired novel object discrimination, denoting deficits in short-term memory (Fig. 6B). Remarkably, anti-ac-tauK174 treatment of TBI mice completely preserved normal memory in this task (Fig. 6B). Upon examining neuropathological changes, mice with TBI displayed significantly elevated levels of ac-tau and t-tau, but not p-tau, by western blot compared to those that received the sham injury (Sup Fig. 3A, B). Clone 1 antibody treatment did not significantly affect levels of ac-tau, p-tau, or total tau (Sup. Fig. 3 C, D). While no marked difference in p-tau was detected, there was a negative correlation between p-tau levels and memory performance with respect to individual animals (Fig. 6C). Iba-1 signal in the lower cortex (LOI 2) also negatively correlated with memory performance (Fig. 6D), as expected since NOR involves the entorhinal cortex [62].

To investigate whether anti-ac-tauK174 antibody could affect pathological tau spreading associated with TBI, we utilized Tau RD P301S FRET biosensor assay [63] and assessed in vivo antibody treatment efficacy in reducing tau seeding activity in vitro (Fig. 6E). Specifically, we seeded Tau RD HEK293T cells with PS19 lysate and observed a significant increase in CFP/YFP FRET signal with TBI and a downward trend with antibody treatment (*p* = 0.0634) (Fig. 6F, G). These findings highlight the functional benefits of anti-ac-tauK174 immunotherapy, which both prevents TBI-induced memory deficits in PS19 mice and reduces tau seeding and spreading in human cells.

To further explore the relevance of lowering ac-tauK174 levels by immunotherapy in humans, we examined whether specific acetylation of tau at K174 is increased in TBI subjects (Sup. Table 3), detected by Clone 2. Notably, plasma ac-tauK174 was increased by 50% within 24 h of TBI, compared to controls (*p* < 0.001), and remained consistently elevated across all time points (Fig. 6H, I, Sup. Fig. 4). Taken together, our findings suggest that ac-tauK174 represents a potential early therapeutic target after TBI that may mitigate the initial inflammatory responses associated with pathological tau acetylation.

## Discussion

Immunotherapy has been increasingly considered as a treatment for tau-mediated neurodegeneration. However, while multiple anti-tau antibodies have been tested in different animal models of disease, human clinical trials have shown poor outcomes [64–66]. These studies however have targeted p-tau or conformation-specific tau, but the field has recently turned to developing antibodies that target other disease-associated modifications of tau, including ac-tauK280 [67]. While tau is to some extent normally acetylated, it becomes more extensively acetylated under pathological conditions [5, 7, 10]. We previously established that acetylation of tau at lysine 174 prevents its degradation and is an early, critical, and pathological change to soluble tau in AD brain [10, 13]. Levels of acetylated tau, detected by a polyclonal antibody, have been previously shown to be elevated acutely in TBI patients, and pharmacologically reducing tau acetylation is neuroprotective in TBI [12]. In this study, we generated and characterized two clones of anti-ac-tauK174 mouse monoclonal antibodies and investigated their potential as a therapeutic for tauopathy alone and in combination with brain injury.

We first demonstrated that weekly IP injection of anti-ac-tauK174 antibody prevented weight loss and abnormalities in hindlimb extension and cognition in aged PS19 mice. We also showed that anti-ac-tauK174 treatment reduced pathogenic tau spread and p-tau accumulation and trended towards a rescue in hippocampal volume loss. Thus, we have shown here that targeting ac-tauK174 in a model of tauopathy improves cognitive function and mitigates tau pathology, consistent with our previous reports using small molecule inhibitors of the tau acetyltransferase p300/CBP [13, 37]. Although the exact mechanism of tau immunotherapy remains elusive, it is proposed that these antibodies can reach the brain and bind to the target tau species in the extracellular space or inside neurons, which could lead to their sequestration, neutralization, and enhanced microglial clearance [25]. The exact mechanism by which anti-ac-tauK174 benefits tauopathy animals warrants further investigation.

TBI increases the risk for tauopathies, including AD [4]. Tau deposition in the brains of AD subjects with TBI history positively correlates with the extent of cognitive decline, CSF-tau, and CSF-amyloid [68]. Genetically reducing tau also mitigated cognitive impairment caused by cortical contusion injury and other TBI models [69]. Besides tauopathy, TBI is characterized by a persistent increase in activated microglia and infiltration of peripherally derived macrophages associated with increases in proinflammatory cytokines [70]. For example, sustained microglial activation has been observed in the brain up to 18 years after TBI in postmortem analyses and by PET scans in TBI subjects [71, 72]. Furthermore, inducing experimental TBI in AD-relevant animal models has been shown to alter inflammatory responses and accelerate cognitive decline [73–75]. The shared pathophysiology of TBI and tauopathy underscores the potential of tau as a therapeutic target for TBI.

Upon TBI, the high strain placed on axons may inhibit the mobility of adjacent microtubules to slide past each other in response to axonal stretching, resulting in long microtubule breakage and short microtubule detachment from microtubule bundles [76]. These mechanical forces at the site of injury promote soluble tau detachment from the microtubule [77, 78], facilitating post-translational modifications at the disease-associated residues and increasing the propensity of tau to aggregate into oligomers and NFTs [79, 80]. While some studies have shown that repeated TBI (such as in CTE) increases tau hyperphosphorylation [81], fairly little is known about changes in the abundance of other forms of pathological tau species upon a single mild injury. Here, we reported a striking increase in ac-tauK174 levels in human plasma early post-TBI, consistent with our previous report [12]. Given the beneficial effects of targeting ac-tauK174 in tau transgenic mice, anti-ac-tauK174 is well-positioned to be a candidate for immunotherapy in tau-mediated neurodegeneration in TBI.

We also demonstrated here that weekly IP injections of an anti-ac-tauK174 antibody prevented TBI-induced memory deficits, and decreased p-tau deposition and microgliosis, in PS19 mice with TBI. snRNA-seq revealed that anti-ac-tauK174 treatment ameliorated aberrant microglial reactivity patterns in PS19 mice with TBI, as demonstrated by the reduction in DAM signature genes (*Tyrobp*, *B2m*, and *C1qa*). Notably, a chronic increase in *C1q* expression coupled with neuron loss and chronic inflammation has been reported after TBI [82, 83], linking *C1q* with constitutive microglial activation in TBI. Further microglia subcluster analysis revealed that anti-ac-tauK174 treatment decreased the TBI-induced cluster associated with cytokine and inflammation transcription regulator downstream genes. Importantly, the most significant upstream regulator was TNF, a prominent proinflammatory cytokine that is upregulated by TBI and toxic to neurons [84, 85]. Being an NF-kb target gene itself, TNF can also activate NF-kb, resulting in a feedforward loop [86].

In addition to chronic neuroinflammation, TBI leads to progressive atrophy of white matter tracts and myelin breakdown in the human brain [87–89]. Here, our snRNA-seq analysis revealed an increase in myelination-related genes (*Mbp, Sox10, Tcf7l2*) with antibody treatment. Interferon (IFN) secretion is markedly increased after TBI, leading to secondary brain damage [90–92]. Interestingly, the IFN-responsive oligodendrocyte population (IRO) signature (*Ifi27l2a, B2m*) and IFN-gamma genes (*Arid5b, Bpgm, Auts2*) decreased in antibody-treated PS19 mice with TBI, suggesting that ac-tauK174 immunotherapy reduces oligodendrocyte interferon-related responses.

In summary, we provided strong evidence of ac-tauK174 immunotherapy in ameliorating tau pathology and glial responses associated with TBI, however, future studies are necessary to fully understand the scope and implications. It is important, for example, to note the limitations associated with using a single transgenic mouse model in our preclinical study. In addition, mice of mixed sex were used for behavioral and immunostaining experiments, while only male mice were included for transcriptomic analyses. The potential role of sex differences in response to the treatment needs further investigation.

## Conclusion

In summary, our findings underscore the critical role of ac-tauK174 in the pathophysiology of tauopathy and TBI. Treatment with anti-ac-tauK174 antibodies yielded promising results in reducing tau pathology, improving cognitive function, and mitigating inflammatory responses. Our study presents evidence for anti-acK174 immunotherapy as a promising therapeutic for the treatment of tauopathy-driven TBI in humans.

### Electronic supplementary material

Below is the link to the electronic supplementary material.

Supplementary Material 1

Supplementary Material 2

Supplementary Material 3

Supplementary Material 4

## Data Availability

The datasets used and/or analyzed during the current study are available from the corresponding author on reasonable request.

## Abbreviations

Ac-tau: Acetylated-tau
Ac-tauK174: Acetylated-tau at lysine 174
AD: Alzheimer’s disease
CTE: Chronic traumatic encephalopathy
DAM: Disease-associated microglia
DAO: Disease-associated oligodendrocyte
DI: Discrimination index
FTLD: Frontotemporal lobar degeneration
hTau: Human tau
IFN: Interferon
IP: Intraperitoneally
IPA: Ingenuity pathway analysis
IRO: Interferon-responsive oligodendrocyte
K_D_: Dissociation constant
KO: Knockout
K174: Lysine residue 174
Mbp: Myelin basic protein
Mo: Month
MWM: Morris water maze
NFTs: Neurofibrillary tangles
NOR: Novel object recognition
p-tau: Phosphorylated-tau
scRNA-seq: single-cell RNA-sequencing
snRNA-seq: single-nuclei RNA-sequencing
SPR: Surface plasmon resonance
TBI: Traumatic brain injury
WT: Wild-type
