# Supplementary material for: Anti-acetylated-tau immunotherapy is neuroprotective in tauopathy and brain injury
Source: Mol Neurodegener. 2024 Jun 24;19:51. doi: 10.1186/s13024-024-00733-9 (PMC11197196; doi:10.1186/s13024-024-00733-9)
Supplement: Supplementary file 4 — Supplementary Material 4 [file 13024_2024_733_MOESM4_ESM.pdf]

Fig. S1, related to Fig. 2.

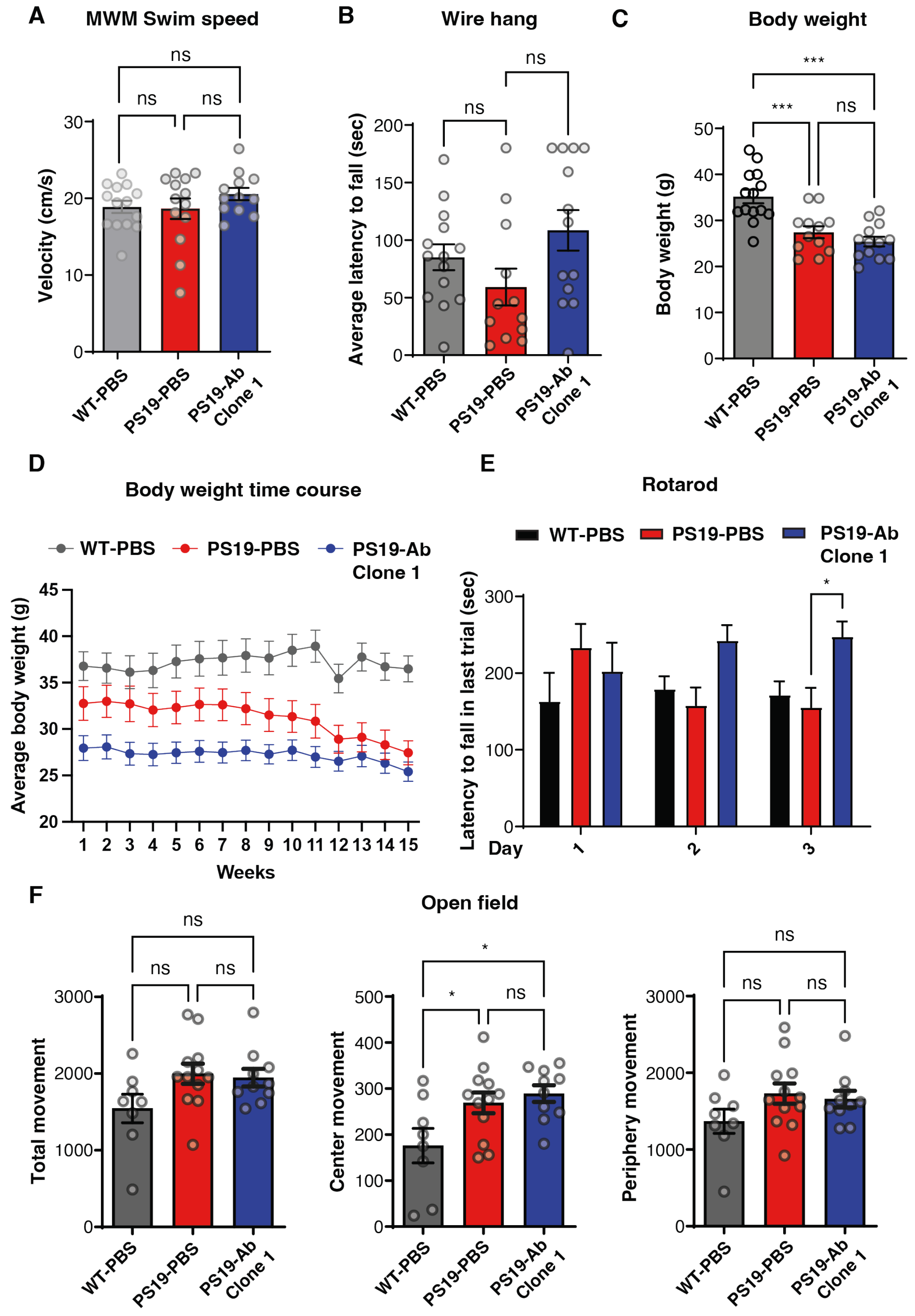

(A) Swim speed in the Morris Water Maze (MWM) 72 hr probe trial. ns, not significant, by one-way ANOVA, Tukey's multiple comparison test. **(B)** Average latency to fall in the wire hang assay. ns, not significant by one-way ANOVA, Tukey's multiple comparison test. **(C)** Body weight of mice at the wire hang assay. ***$p<0.001$, by two-way ANOVA, Tukey's multiple comparison test. **(D)** Average body weight of three groups of animals throughout the course (15 weeks) of the experiment. WT-PBS: n=12, PS19-PBS: n=14, PS19-Ab: n=14. Error bars: SEM. The dip at week 12 was due to MWM. **(E)** Average latency to fall in the last trial of the rotarod assay by day. *$p<0.05$, by two-way ANOVA, Tukey's multiple comparison test. **(F)** Average total movement, center movement, and periphery movement in the open field assay. ns, not significant, *$p<0.05$, by one-way ANOVA, Tukey's multiple comparison test.

Fig. S2, related to Fig. 5.

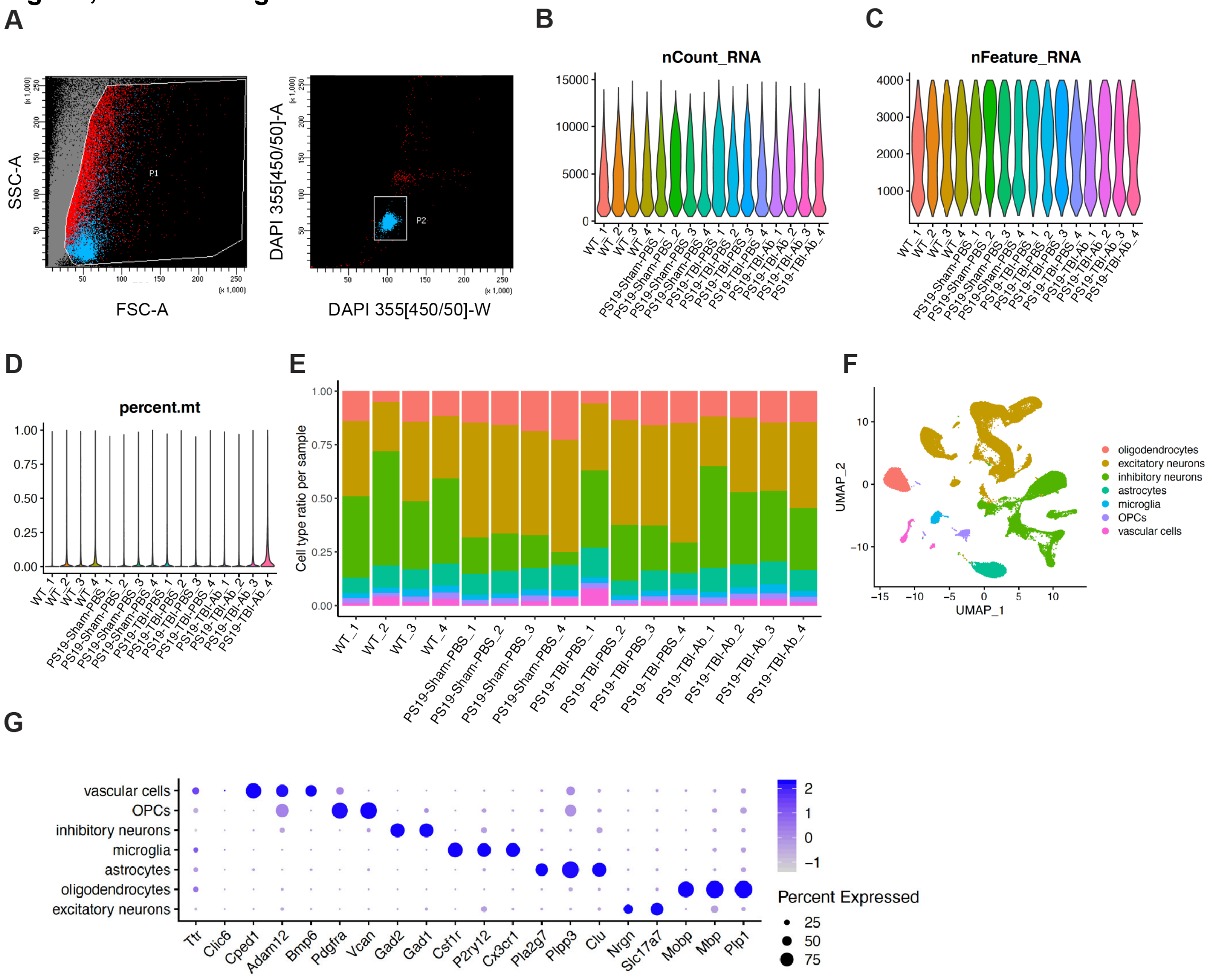

(A) FACS gating strategy for sorting singlet, DAPI-stained nuclei. **(B-D)** Violin plots showing spread of total genes (B), total features (C), and percent of mitochondrial genes (D) detected per nuclei for each individual sample. **(E)** Proportion of cell types for each individual sample. **(F)** UMAP plot of all single nuclei and their annotated cell types. OPCs: Oligodendrocyte progenitor cells. **(G)** Percentage and average expression levels of maker genes for indicated cell types.

Fig. S3, related to Fig. 6.

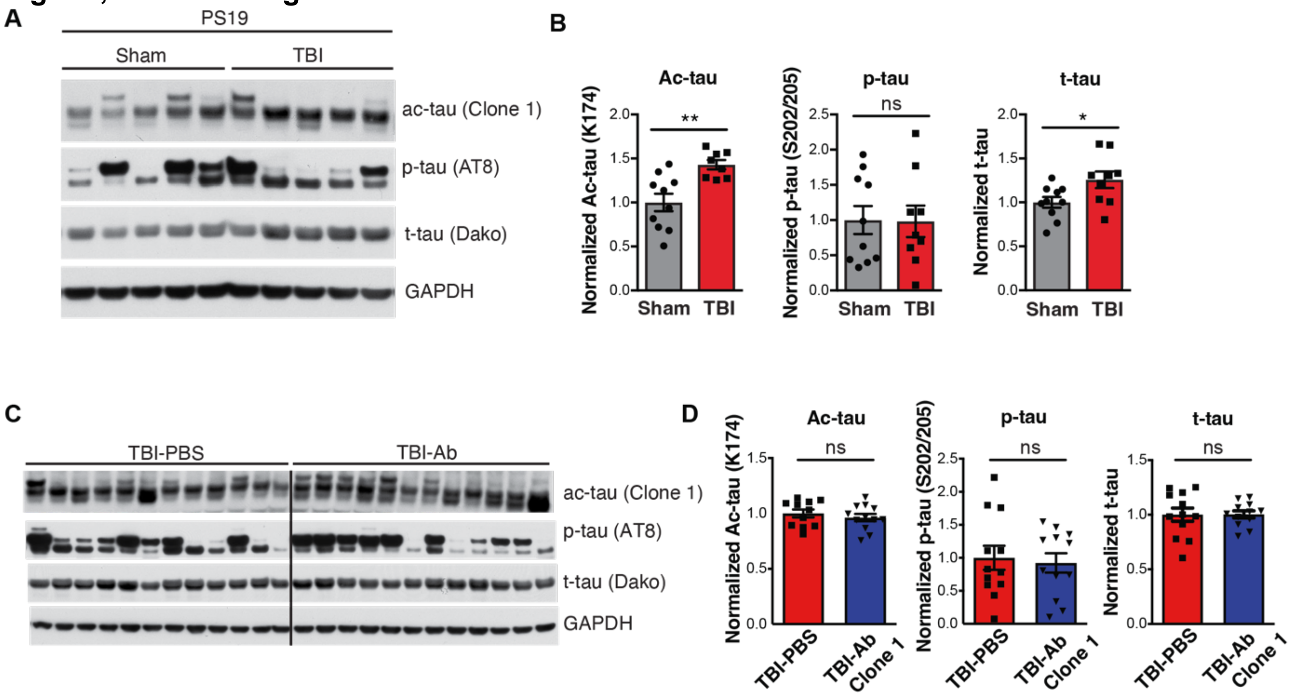

(A) Representative immunoblot of acetylated tau (Clone 1), p-tau (AT8), total tau (t-tau, Dako) and GAPDH in cortex lysate of PS19 mice subjected to TBI and sham surgery. **(B)** Quantification of levels of ac-tau, p-tau, t-tau relative to GAPDH and normalized to sham group. n=10 (sham), n=9 (TBI). *p<0.05, **p<0.01, by unpaired t-test. **(C)** Representative immunoblot of acetylated tau (Clone 1), p-tau (AT8), total tau (t-tau, Dako) and GAPDH in cortex lysate of PS19 mice subjected to TBI and treated with PBS or Clone 1 Ab. **(D)** Quantification of levels of ac-tau, p-tau, t-tau relative to GAPDH and normalized to TBI-PBS group. n=12 per group. ns, not significant, by unpaired t-test.

Fig. S4, related to Fig. 6.

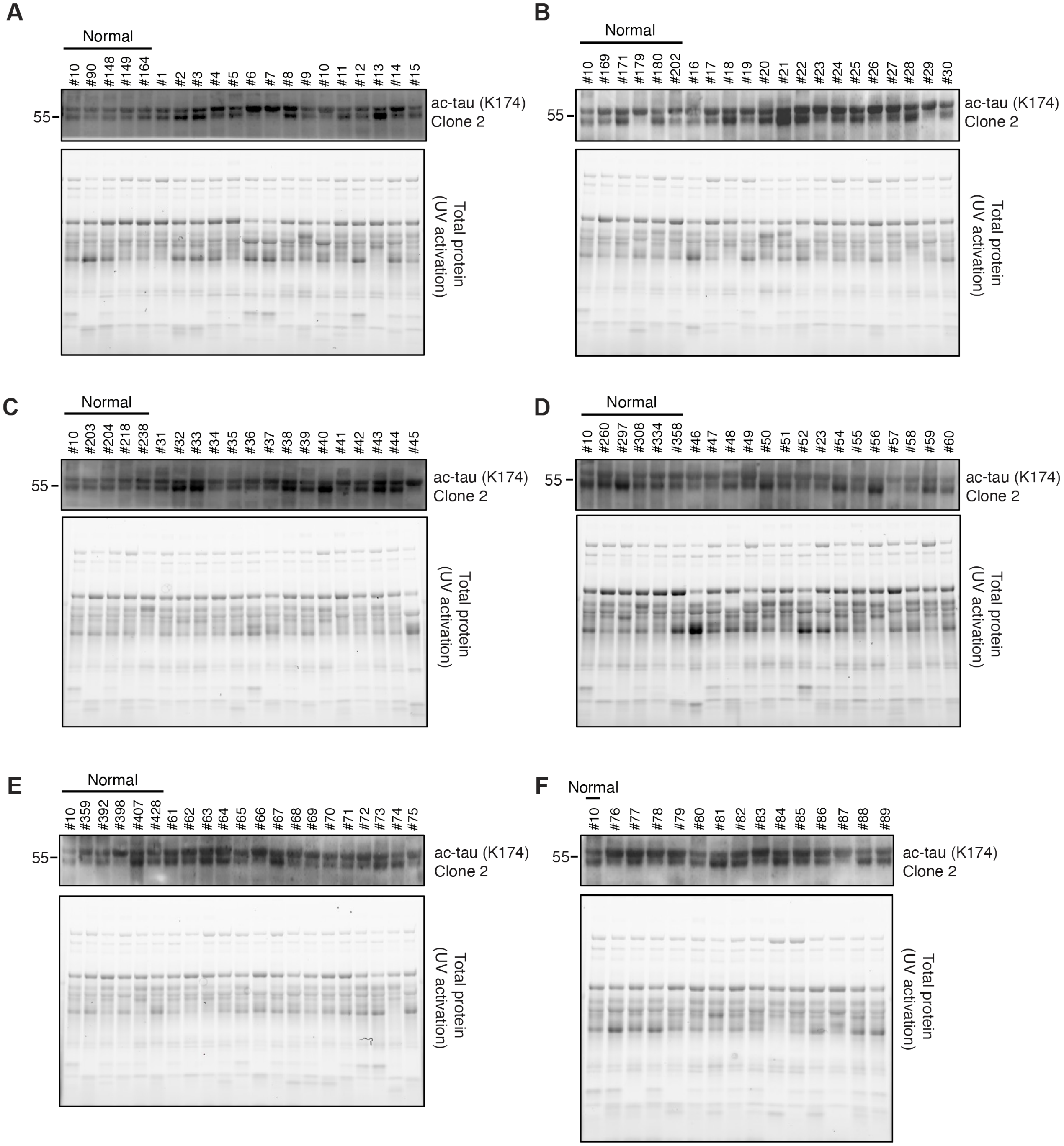

(A-F) Immunoblots of acetylated tau (K174) (Clone 2) in control (Normal) and TBI human plasma samples: (A) TBI #1-15, (B) TBI #16-30, (C) TBI #31-45, (D) TBI #46-60, (E) TBI #61-75, (F) TBI #76-89.
